# A Group Therapeutic Songwriting Intervention for Family Caregivers of People Living With Dementia: A Feasibility Study With Thematic Analysis

**DOI:** 10.3389/fmed.2018.00151

**Published:** 2018-05-22

**Authors:** Felicity A. Baker, Phoebe Stretton-Smith, Imogen N. Clark, Jeanette Tamplin, Young-Eun C. Lee

**Affiliations:** Faculty of Fine Arts and Music, University of Melbourne, Melbourne, VIC, Australia

**Keywords:** family caregivers, dementia, music therapy, songwriting, depression, thematic analysis, feasibility

## Abstract

This study aimed to test the feasibility of implementing a group songwriting program with family caregivers (FCGs) of people living with dementia. Fourteen FCGs consented to participate in either the songwriting group (*n* = 8) or control condition (*n* = 6). Participants completed baseline and 7-week measures of depression (PHQ-9), perceptions of their caregiving experience (PACQ), and perceptions of their relationship with the care recipient (QCPR). A six-session group songwriting program was implemented across two sites, focusing on participants co-creating a song about their caregiving experiences. Participation and retention rates were high suggesting the intervention was acceptable. An observed pre-post effect size for the PHQ-9 in the experimental group (*d* = 0.64) and control group (*d* = −0.33) suggests the measure is sensitive to change over a short period of time in this population and has the potential to detect significant change in a larger controlled trial. Qualitative analysis of focus group interviews suggested the songwriting process allowed participants to share their entire caregiver journey with others, differentiating the intervention from standard carer support groups. Participants described group songwriting as enabling them to find connections with other caregivers, create a group identity, and gain insight into their carer journey, subsequently leading to the development of inner strength and personal growth. Qualitative findings suggest coping may be a more relevant construct to measure than caregiver-patient relationship quality or caregivers' perception of caregiving.

## Introduction

### Family caregivers of people with dementia

The current global population of people with dementia (PWD) is 47.5 million and this figure is exponentially rising (1). By 2030, the number of PWD is estimated to exceed 135 million, presenting further significant personal, social, and economic impacts for individuals, families, and society as a whole (1). In Australia, up to 70% of PWD live in the community, often relying on formal and informal assistance to meet their care needs (2). Family caregivers (FCGs) play a vital role in providing informal assistance with reports indicating that 81% of co-resident primary carers provide over 40 h of care for their family member with dementia every week (2). Family caregivers are most often the PWD's spouse (42%) or adult child (44%), and figures suggest that men are almost twice as likely as women to exclusively rely on informal assistance and care (2). Through taking on this role, FCGs ensure PWD remain at home for as long as possible. This not only directly benefits the PWD, but represents a significant economic contribution to society.

The dual nature of caregiving encompasses a range of burdens and rewards to those providing informal care (3). While there are satisfying, positive, and rewarding aspects to the caregiver role (3, 4), the tasks and level of burden associated with caregiving mean many FCGs need support (5). In many cases, the demands of caring can override FCGs' capacity to cope, leading to negative health and psychosocial outcomes, such as depression, anxiety, fatigue, and illness (6, 7). A recent review exploring the impact of caregiving found FCGs of PWD experience a number of specific challenges affecting their ability to cope (8). These include personal, social, and emotional challenges, such as identity change, isolation, and feelings of anger, fear, embarrassment, guilt, confusion, frustration, sorrow, and grief (8). In addition, FCGs were found to experience stress and burden associated with the deteriorating relationship and ability to communicate with the PWD, managing symptoms and behaviors of the PWD, and taking on additional domestic roles and challenges (8). Despite these impacts and challenges, research indicates that FCGs often underutilize a range of existing resources and support services to cope with the caregiving role (9). This underutilization has been linked to challenges and influences associated with building a caregiver identity (9). This includes associated roles and responsibilities, and the impact of these on available time and energy for FCGs to engage in activities and interventions that may help shape a carer role identity (9).

FCGs may have difficulty transitioning into a carer role identity, both initially and with subsequent changes in the caregiver context (10). Transitions to, and within, the FCG role are entwined with existing roles and relationships, family values, and social and cultural norms, as well as the FCGs personal beliefs and self-expectations about their responsibility to provide care (10). For example, FCGs often present with a self-expectation or altruistic tendency to focus their energy solely on the care recipient and in doing so neglect their own need to rest and engage in social and leisure activities (11, 12). Comparative studies have found that FCGs stress levels are exacerbated as new care demands arise or existing care demands amplify with disease progression (11–14). Further, frequent personality changes and unpredictable or uncharacteristic behaviors associated with dementia can be distressing for FCGs as they attempt to adapt or modify their strategies to cope with changing care demands (1, 15). As the needs and dependency of the care recipient increase, the caregiver/care recipient relationship and context changes, and the FCG is faced with further transitions in role and identity (9, 10). While supporting FCGs is an increasing focus of practice, there is a need for further research into potential practical interventions that are both acceptable and accessible.

In a systematic review of interventions for FCGs of PWD, Elvish et al. (16) found that the majority of studies employed a cognitive-behavioral approach (17) and/or incorporated stress and coping theoretical models (18). Additionally, Elvish et al. (16) found that seven of the eight studies employing psychoeducational-skill building produced significant results. Psychoeducational skill building includes coping and managing emotional difficulties arising as a primary consequence of caring for a PWD (16). The viability of this method was reflected in the interventions' impact on FCGs' levels of depression, anxiety, wellbeing, quality of life, and attitudes toward caregiving (16). However, many of these reported interventions required a heavy attendance commitment (at least 6 weeks for 2-h at a time). When caring for a PWD, organizing alternative care for such an extended period of time and for consecutive weeks may impact attendance and, in turn, the potential impact of the intervention. In the same review, Elvish et al. (16) found that psychotherapy-counseling sessions were only present in one study and no quantitative measures were included to capture its impact. Notably, the authors concluded that interventions should focus on tailoring the content for individuals or, alternatively, create group interventions based on a common issue. This is supported by other studies highlighting the need for interventions that recognize the unique and idiosyncratic nature of family caregiving, and diversity and variability between caregiver experiences, needs, and contexts (8, 10).

### Music therapy interventions for family caregivers: overview and evidence to date

There is increasing interest in exploring how music therapy approaches can assist FCGs to cope with their caregiving roles. A number of studies have examined interventions designed to support both the FCG and care recipient. Clair (19) and Clair et al. (20) utilized music therapy programs to support the maintenance of relationships between FCGs and PWD. These studies found that, by increasing the alertness of PWD through music, PWD and their families were able to share meaningful musical experiences. While these findings support the use of music therapy to enhance the quality of the FCG/PWD relationship, focus was not given to the FCG's individual wellbeing. Baker et al. (21) developed a home-based training program that spousal carers of PWD could learn to use with the person being cared for. The focus of the intervention was three-fold: (1) to maintain reciprocity between FCG and care recipient, (2) to address FCG sense of burden, and (3) to calm the PWD during periods of anxiety and agitation. Although a small sample size precluded quantitative measures being meaningful, qualitative analysis of FCG's diary entries indicated that engaging in music enhanced enjoyment, relaxation and quality of relationship, strengthened reciprocity, and increased satisfaction with the caregiving role (21). Other research has reported benefits of interventions designed solely for FCGs.

Brotons and Marti (22) used various music therapy-oriented support groups with FCGs where the care recipients were not present. Their study enabled participants to disclose uncomfortable thoughts and feelings while simultaneously garnering support from other FCGs (22). Similarly, O'Kelly (23) used individual songwriting with a female spouse-carer of a man with motor neuron disease. The songwriting process enabled the expression of feelings of burden, depression, and guilt while also reconciling conflicting emotions (23). Psychoeducational songwriting approaches were utilized in Klein and Silverman's (24) study with FCGs of PWD. Here, the songwriting process was used to foster healthy coping skills. Drawing on a predefined outcome-oriented model of songwriting (25), Klein and Silverman (24) concluded that the songwriting approach distracted participants from their ongoing stress, reinforced messages of healthy coping, and enabled them to gain insight into themselves and their context.

### Group therapeutic songwriting: overview and evidence to date

Therapeutic songwriting is defined as the process of creating, notating, and/or recording lyrics and music within a therapeutic relationship to address psychosocial, emotional, cognitive, and communicative needs of a client or group of clients (26). Songwriting is widely employed by music therapy clinicians worldwide (27, 28) with a diverse range of clinical populations (25). Research has documented therapeutic songwriting interventions with children, adolescents, adults, older adults, and those at end of life in both acute and community settings (25). Theory-informed approaches are emerging (25, 29, 30) and there is growing evidence to suggest that, when used in group therapy contexts, songwriting improves health and wellbeing indicators for depression, anxiety, and quality of life [e.g., (31)].

There is increasing research to support the use of group songwriting with older adults, both those with declining cognition (32–34), and those with intact cognition (35). For people living with dementia, group songwriting has been found to have positive impacts on language function, orientation, and memory (32, 34), social interaction and cohesion (33), motivation, belonging connectedness, and feelings of accomplishment and ability (34). Similarly, in a study of healthy older adults residing in a retirement village, group songwriting enabled participants to feel connected, experience feelings of accomplishment, and derive meaning (35). Studies here indicate that group songwriting fosters connection, belonging and group cohesion although empirical studies are yet to confirm this (36). Further, no quantitative studies with older adults have explored the degree of change in wellbeing consequential to group songwriting processes.

More recently, Baker and Yeates (37) reported on a pilot study of a songwriting protocol developed for FCGs of PWD who were attending a dementia care day center program. Over three sessions, four FCG participants (two spouses and two adult children) created and recorded a single song that reflected their shared experiences of caregiving—both the positive and challenging. Participants were interviewed post-intervention and an interpretative phenomenological analysis of transcripts showed that FCGs' experiences went beyond their expectations. Participants described the collaborative component of co-creating a song as meaningful and empowering, and enabling them to gain insight into themselves, each other, and the caregiver journey through the process (37). Importantly, participants felt that the three-session program was too short and suggested six sessions might enable them to sit for longer with their feelings and shared experiences, thereby enhancing the transformative potential of the group songwriting process (37).

The current study intended to build on the pilot study by Baker and Yeates (37) by extending the songwriting intervention to six-sessions and including both quantitative and qualitative data to better understand the impact of the intervention for participants. Further, this study sought to assess the feasibility of the method and intervention for a future larger scale trial and collect qualitative data to inform the construction of a preliminary theory of mechanisms underpinning any impact on wellbeing.

## Methods

### Trial design

The exploratory pilot and feasibility study was designed as a simple pre-post test design, examining the uptake of group songwriting and testing the acceptability and appropriateness of the measures for FCGs of people living with dementia. The trial included post intervention focus group interviews to better understand participants' experiences of the intervention, enable further refining of the intervention, and identify any perceived benefits or limitations of the study. More specifically, the purpose of the pilot study was to:

1. Determine participation rates and retention rates of FCGs involved in the group therapeutic songwriting process, and whether participants in the control group would be willing to complete the measures despite not receiving the intervention;
2. Provide estimates of the effect of the intervention on levels of depression, and quality of the FCG-PWD relationship in order to ascertain whether these measures might be sensitive to change in a fully powered trial.
3. Assess the acceptability of the intervention and impact of treatment intervention as perceived by carers through focus group discussions.
4. Construct a substantive theory of the processes activated during the group therapeutic songwriting program that could be used to inform larger experimental studies.

As a feasibility study, consenting participants were able to choose either to participate in the songwriting group or to participate in trialing the measures without receiving the intervention. Quantitative data was collected prior to the commencement of the intervention and in the week following completion of the intervention (week 7).

### Participants

Family caregivers were recruited for the study through two dementia care day centers in Melbourne. Both centers are not for profit organizations and their services include day respite and recreational programs for people living with dementia, as well as support groups and initiatives for caregivers living in the community. All participants were caring for family members with dementia who were accessing services at one of the two day centers. Family caregivers were approached by day center staff either in person or by phone to be invited to participate in the study. Participants were eligible if they: (a) were a primary FCGs of a PWD; (b) were caring for a PWD living in their own home; (c) had functional hearing; and (d) could speak English. The FCG relationship to the PWD could be either a spouse, close family member (e.g., child of the PWD), or friend. English could be the participants primary or non-primary language.

In total, 14 FCGs (nine female, five male, aged *M* = 72.29 years, *SD* = 6.72 years) were recruited for this study (Table 1). Eight participants (six female, two male, aged *M* = 70.4 years, *SD* = 6.0 years) consented to participate in the experimental group, and six participants (three female, three male, aged *M* = 74.8 years, *SD* = 6.9 years) consented to participate in the standard care group. Across both conditions, the vast majority of FCGs were caring for their spouse (*n* = 11), while two participants were adult children caring for their parent, and one participant was caring for her sibling. Although 64.3% (*n* = 9) of all FCGs recruited were female, the care-recipients of participants were equally male and female.

**Table 1:** Participant characteristics.

**Group**	**Participant pseudonym**	**Age**	**Gender**	**FCG/PWD relationship**
EG (*n* = 8)	Charles	74 y/o	Male	Spouse caring for wife
	Michelle	64 y/o	Female	Adult child caring for mother
	Lotte	75 y/o	Female	Spouse caring for husband
	Julie	72 y/o	Female	Sister caring for brother
	Alison	66 y/o	Female	Spouse caring for husband
	Tony	80 y/o	Male	Spouse caring for wife
	Lisa	61 y/o	Female	Spouse caring for husband
	Anne	71 y/o	Female	Spouse caring for husband
SC (*n* = 6)	Edith	85 y/o	Female	Spouse caring for husband
	Mandy	66 y/o	Female	Adult child caring for mother
	George	80 y/o	Male	Spouse caring for wife
	Vincent	76 y/o	Male	Spouse caring for wife
	Sam	72 y/o	Male	Spouse caring for wife
	Roslyn	70 y/o	Female	Spouse caring for husband

*EG, Experimental Group; SC, Standard Care*.

From the experimental condition, two songwriting groups were established (one at each site). At site 1, five FCGs participated in group songwriting—three female spouses, one male spouse, and one female sibling. At site 2, three FCGs participated in group songwriting—one female spouse, one male spouse, and one adult daughter. Participants in the songwriting groups were predominantly from English-speaking backgrounds, however, some participants were born outside of Australia (England and Europe) and one participant had English as a second language.

### Theoretical basis and description of intervention

Family caregivers participated in six weekly 1-h songwriting sessions facilitated by a trained music therapist (author 2). Sessions took place in private rooms at the dementia care day centers and group size ranged from three to five participants. The day center manager was also present in sessions at one of the sites, providing additional support and assisting the therapist when needed. In this intervention, both groups dedicated the six sessions to creating and recording one song. This allowed participants to explore their experiences of caregiving in-depth, and engage deeply in the creative process.

This intervention was framed by an introduction session (week one) and recording session (week six). Session one comprised a general introduction, designed to provide space for each participant to share their story of caring for a PWD and to build trust and rapport. Session six was spent refining, rehearsing, and recording the song. In sessions two to five, the process of original songwriting (25) was largely guided by participants and moved cyclically between sharing of individual stories, finding commonalities between experiences, and developing ways to tell the collective story of the group. This occurred through: brainstorming (brainstorming ideas, identifying common aspects to the FCGs experiences); lyric creation (constructing a chorus that captured the overall message of the song and verses that explored the shared experiences of the group); and music creation (creating music that supported the feelings expressed in the lyrics). Although the music therapist was predominantly responsible for music creation, participants were encouraged to be involved at numerous stages of the process. This included discussing musical preferences, suggesting style of music for the song creation, and making musical choices about melodies, harmonies, and accompaniment styles.

This intervention was formulated on the basis of the researchers' assumptions: that a targeted group songwriting protocol would enable FCGs to explore their perceived caregiving experience, reflect on their relationship with the care recipient, express positive feelings, and release negative feelings, subsequently leading to reduction of mood symptoms. The intervention drew on a combination of insight-oriented, strengths-oriented, narrative, and cognitive reframing therapeutic songwriting frameworks (25, 29). This aimed to support FCGs voice their experiences, explore issues around changing roles and identity, develop insight into sources and consequences of stress, and resolve conflicting feelings about their situation (29). Through this process, FCGs were able to break down barriers to coping and wellbeing and recognize their own strengths, abilities, and competencies as carers. The therapeutic approach incorporated specific strategies to address stress and coping (18), particularly emotion-focused approaches (self-expression, cognitive-reframing, validation), and problem-focused approaches (managing difficult behaviors or social situations, self-care). For example, as FCGs expressed maladaptive, self-defeating, or distressing thoughts about their own competencies, the music therapist gently challenged these perspectives and helped them to cognitively reframe these thoughts into constructive positive lyrics (38). Within the program, the therapist stimulated group discussions and helped FCGs transform their ideas and experiences into meaningful lyrics. As the FCGs discussed issues most salient to them, they engaged in the co-construction of lyrics that reflected their shared struggles and joys. Metaphors and imagery were often used to capture the essence of their experiences.

### Outcome measures

The feasibility of outcome measures was determined by collecting data pre and post intervention (at baseline and week 7). All participants completed self-report scales selected to measure levels of depression and quality of relationship between FCG and PWD. We examined completion rates of measures and dropout (non-completion of measures).

### Depression

As this study focused on depression as the primary outcome measure, the *Patient Health Questionnaire* [PhQ-9; (39)] was used to measure symptoms and severity of depression in FCGs. The *PHQ-9* demonstrates acceptable sensitivity and specificity across diverse patient samples and has been found to be sensitive to change in other music therapy studies (40). Each item on the 9-item self-rating scale is scored from 0 (not at all) to 3 (nearly every day). Total scores range from 0 to 27, with higher scores indicating higher levels of depression (e.g., scores between 15 and 19 are indicative of moderately severe depression, and 20–27 indicates severe depression). *PHQ-9* has been found to have good internal reliability (Cronbach's α of 0.86–0.89) and convergent validity as measured against the SF-20 Health-related Quality of Life Scale (*p* < 0.05 for most pairwise comparisons) (39).

### FCG/PWD relationship

Secondary outcome measures comprised two scales measuring quality of FCG/PWD relationship, and positive aspects of caring. These scales were chosen based on their emphasis on psychosocial dimensions of wellbeing, as well as specificity to caregivers. The *Quality of the Caregiver Patient Relationship* [QCPR; (41)] is a 14-item questionnaire measuring the quality of relationship between the PWD and FCG. Items are scored using a 5-point Likert Scale, with higher scores indicating a higher quality of relationship. From the 14 items, 6 items are written and scored in reverse. QCPR has demonstrated acceptable internal consistency (α = 0.82) and concurrent validity. The *Positive Aspect of Caregiving Questionnaire* [PACQ; (42)] is an 11-item questionnaire measuring FCGs' mental/affective state in relation to the caregiving experience. The PACQ utilizes a 5-point Likert Scale for all item responses. Scores range from 11 to 55, with higher scores indicating more positive self-perceptions of caregiving. The PACQ demonstrates acceptable reliability and validity among FCGs of PWD (43, 44).

### Focus group interviews

Family caregivers who participated in the group songwriting sessions were invited to participate in a focus group facilitated by either author 1 or 2 (one held at each study site location). These were audio-recorded and transcribed by the researchers for analysis. Family caregivers were guided to share their experiences of creating songs with other FCGs, including how they felt as they collaborated on a shared theme, and what was meaningful (if anything) about the songwriting process. Focus group interviews were 43 and 46 min in duration. One participant, who was unable to attend the focus group, consented to be interviewed by phone. This interview was conducted by author 2, included the same questions as used in the focus groups, and was 11 min in duration.

### Analysis

Participation rates and completion rates were determined by comparing eligibility, enrolment, intervention completion, and follow-up numbers and transformed into percentages. Appropriateness of outcome measures were determined by examining response rates. Furthermore, differences in pre-post measures across participants were analyzed to ascertain whether the measures were sensitive to change.

The acceptability of the intervention was determined by examining drop-out rates in the experimental group and comments made in the focus group transcripts. As this feasibility/pilot study recruited small numbers to test the sensitivity and suitability of the outcome measures and the acceptability of the intervention protocol, the project was never designed to be adequately powered to test between group differences. However, we calculated effect sizes and associated confidence intervals to determine the size and direction of the effect (45, 46). Standardized mean differences, bias corrected (Cohen's *d*) were calculated for depression, relationship quality, and caregiving experience, with effect sizes categorized as small (*d* ≥ 0.2), moderate (*d* ≥ 0.5), and large (*d* ≥ 0.8) (47). Intention to treat principles were applied to all analyses, meaning that all available data were analyzed as allocated (48).

Focus group data were used as the basis of an inductive thematic analysis, as informed by Braun and Clarke (49). Thematic Analysis is a flexible and recursive process of qualitative data analysis with the overall aim of finding “repeated patterns of meaning” [(49), p.15]. This study utilized an inductive approach in that the analysis process was driven by the data rather than the researcher's theoretical interest or analytic preconceptions regarding therapeutic songwriting with FCGs of PWD (49). This approach was applied within a realist theoretical framework, which aimed to report “experiences, meaning and the reality” of participants [(49), p.9].

Braun and Clarke's (49) six phases of thematic analysis were used to guide the process. Author 1 took primary responsibility for analysis and began by reading and rereading the transcripts and recording initial ideas. Phase 2 of the analysis involved generating initial codes using MAXQDA12 (50) qualitative analysis software. This entailed systematically coding interesting features of each interview transcript and arranging transcript data relevant to each code. Phases three to five involved searching for, reviewing, defining, and naming themes. This process was in part collaborative and involved input from all investigators. Author 1 took primary responsibility for organizing codes into potential themes and assembling relevant data to accompany each theme. These were then sent to the other investigators for comment and feedback, which informed subsequent ongoing analysis and refinement by Author 1. When reviewing the themes, researchers considered whether the themes worked in connection with both the accompanying coded extracts and the interview transcripts as a whole. Some changes to the initial themes were made during this phase, including combining two themes into a larger broader theme. In the final phase (producing the report), extracts from the transcripts were selected and further analyzed to illuminate the findings. This was again an ongoing and recursive process occurring while the report was drafted, revised and finalized. In addition to thematic analysis of the interview transcripts, Author 1 examined the connections between themes and constructed a framework to explain how group songwriting led to identified changes in FCGs. This proposed explanatory framework was revised in collaboration with the other investigators.

## Results

**Figure 1:**
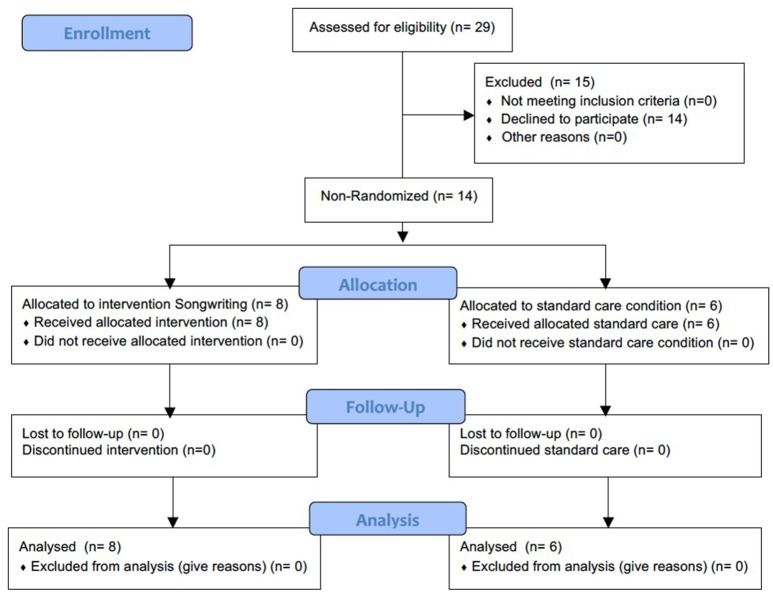
Consort Flowchart for recruitment, participation, and retention rates.

Figure 1 illustrates the CONSORT flowchart for recruitment, participation and retention rates. In this study, participation rates were 48.3% (*n* = 14) and retention rates were 100%.

### Appropriateness of outcome measures and estimates of the effects of the intervention

A moderate effect size was observed for the pre-post scores on the PHQ-9 for the Experimental Group (*d* = 0.64; 95% CI = −0.40, 1.61) (Table 2). In contrast, a small effect size was observed for the pre-post scores on the PHQ-9 for the Standard Care group (*d* = −0.33; 95% CI = −1.47, 0.81). The results suggest that depression is a valid variable to measure and that the PHQ-9 was sensitive to change capturing differences over a short period of time. Other measures did not appear to capture changes in quality of the caregiver-recipient relationship nor changes in positive aspects of caregiving. Missing data were minimal. In the PHQ-9, one item in two participants' measures were missing in the pre-test and one item from one participant's post-test. For the QCPR, one person omitted three items in the pre-test, 2 people omitted one item in the pre-test, and none in the post-test. For the PACQ, one person omitted one item in the pre-test and none in the post-test.

**Table 2:** Means and SD for outcome measures.

	**EG (*****n*** = **8)**		**SC (*****n*** = **6)**	
	**Pre**	**Post**	**Pre**	**Post**
PHQ-9	5.2 (5.3)	2.6 (2.2)	1.8 (1.7)	2.5 (2.2)
QCPR	55.1 (7.6)	56.1 (6.3)	45.7 (7.6)	51.0 (10.8)
PACQ	39.3 (7.7)	36.9 (11.6)	34.9 (12.1)	32.9 (10.1)

*EG, experimental group; SC, standard care; PHQ-9, Patient Health Questionnaire-9; QCPR, Quality of Caregiver Patient Relationship; PACQ, Positive Aspects of Caregiving*.

### Acceptability of the intervention

Attendance rates were strong for all songwriting participants suggesting that FCGs considered the intervention acceptable. FCGs attended an average of 5.5 songwriting sessions [91.7%], *SD* = 0.71) from a maximum of six sessions.

### Participants' perceptions of the impact of group therapeutic songwriting on their wellbeing

Six themes were developed through inductive thematic analysis of interview transcripts. The songwriting process was found to enable participants to: (1) share the whole carer journey; (2) find connections with other FCGs; (3) gain clarity around the carer journey; and (4) develop group identity. Group therapeutic songwriting was also found to: (5) foster inner strength and personal growth; and (6) fill a gap not met by other support groups. Each of these themes are described in detail below.

### Theme 1. sharing the whole carer journey: from woe to go

Participants described the group songwriting program as unique and helpful in that it allowed them to share their whole caregiver story with other FCGs. The concept of the “whole” caregiver story encompassed having the opportunity to talk about both the stages of caregiving (pre-diagnosis, diagnosis, and post-diagnosis), as well as different aspects of the caregiver experience. For example, **Alison** commented that the songwriting program enabled participants to explore and express the *emotional* journey of being a FCG; from “not knowing” to “emotional discovery” to trying to “find an answer” and “going forward.” She states:

In this session, we talked more about our emotions and the journey that emotionally we've been on. From the problems of even not knowing what we were dealing with through the emotional discovery of this diagnosis of dementia and what it meant, and then the road to finding an answer, to going forward. So we talked about that whole journey from woe to go, which isn't something that you do in a carer support group

As well as having the opportunity to share their own carer journeys, participants highlighted the value of hearing other carers' stories and struggles. **Charles** and **Lisa** commented that it was a positive and interesting experience, “listening to other people, their problems or difficulties” (Charles) and “hearing other people's stories and how different [everybody's journey is]” (Lisa). **Alison** added that the opportunity to hear other FCGs' talk about their experiences was “enlightening,” while **Lotte** described it as therapeutic. Further, the process of songwriting involved encapsulating these whole carer journeys into meaningful song lyrics, prompting participants to think and share alongside other FCGs.

**Lisa:** I'd encourage them to do it [group songwriting] and say it's challenging and gets your thoughts thinking and sharing with other people in the same position actually, you know, putting the words onto paper. And it does, um…

**Julie:** Lights up your brain.

**Lisa:** Yeah, yeah, definitely. And it's just a word on a board. And even though it's just a word, there's so much behind those words.

### Theme 2. we're singing the same tune: finding connections

Five participants (Anne, Charles, Alison, Michelle, Lotte) commented on the importance and impact of finding connections and points of meeting with each other during the group songwriting experience. Finding a common thread was identified as a pivotal experience for the groups, helping to foster a sense of connectedness and reduce a sense of loneliness. **Lotte** commented on the value of being able to participate in a group with other FCGs of PWD and how this afforded her opportunities to share her problems, receive empathy and feel less alone. This in turn enhanced her ability to cope with the caregiver role. She recounts:

**Lotte:** Your friends drop off somewhat…I felt terribly, terribly alone at the early part [of taking on a FCG role] where I couldn't participate…I've only got two friends that will listen to me occasionally when I'm down. But generally, people don't want to know when you're down, right? Well, with the other carers, you're not so intimidated or shy or whatever you want to call it. You know they've got problems too and everybody talks about their problems. And so, you know, you don't feel so alone, then.

**Charles:** There's empathy there.

**Lotte:** Yes, yes. That loneliness to me doesn't matter that much anymore now that I've talked to other people. I know others are in the same boat. So yeah, you cope better then.

Particular aspects of the songwriting process also prompted participants to identify common threads and find points of meeting and agreement. **Lotte** articulated the process of moving from sharing individual experiences to creating lyrics that reflected the shared experiences of the group: “We communicated with each other and talked about our experiences individually…[then] we got to a point where we accept, well that's important to all three of us so that's got to go in the song. It's not only important to me.” Further, participants found that coming to an agreement on the song's direction fostered feelings of group cohesion. **Alison** and **Lisa** articulated that in this stage of the songwriting process it felt like participants “were all contributing…it was more cohesive” (Alison) and that “rather than just ‘you do your bit, I’ll do my bit', we all did it together” (Lisa). **Anne** talked about how this was “solidifying” for the group. She states:

It was very much solidifying. And we were all kind of, yeah, we were singing the same tune obviously. We found an agreement and a feeling of where we would be going with the song. So I think, yeah, that did give us that feeling of cohesion.

### Theme 3. gaining clarity around the carer journey: discovering we've already found a way through

Caregivers found that the songwriting process enabled them to gain clarity and insight into how well they were actually coping, often with quite difficult circumstances, and “discover that [they'd] already found a way through.” Alison and Anne found that the songwriting process enabled them to gain clarity around where the carer journey was heading. When **Anne** was asked why she agreed to participate in the program, she relayed that she “was prepared to keep an open mind and try anything.” Having concerns about not knowing “where to go” or “what to do,” Anne was thankful the songwriting process helped her to “clarify in my own mind where we're going, and the reality of it.” Similarly, Alison articulated that the songwriting process enabled exploration and consolidation of various aspects of the caregiver experience, reinforcing her perception of her ability to cope. Asked how she would describe reasons for taking part in a songwriting group to other FCGs, Alison states:

**Alison:** Okay, let me think. I would say, it [the songwriting group] will give you the opportunity to explore lots of aspects of what you're dealing with and really just discover that you've already found a way through, but just coalesce to it…

This process of clarification, reinforcement, and discovery regarding the caregiver journey was attributed to aspects of the group songwriting process, specifically. **Alison** reflected that the creative process of lyric and music creation prompted participants to identify positive, as well as challenging aspects of the caregiver experience. This led to FCGs identifying ways they cope and “deal” with the role. She states:

I think the fact that you were asking us to draw on these emotions and put them in words—words that could then be put to music and song. Again, put a different perspective on it. So it made us look for the positive rather than just the negative…It was trying to look at some of the good things that have come out of it and how we have learned to deal with those issues.“

Participants also highlighted the group context as a key element in clarifying their individual carer journeys. During the group songwriting process, participants heard other FCGs' stories and were able to put their own experience into context. **Alison** and **Lisa** recognized this.

**Alison:** I agree with Lisa, there were so many different people that had had different aspects and different experiences [of caregiving] and it put your experience into context.

Finally, the lyrical representation of this theme (“discovering we've found a way through”) was an element of the final product (the song creations) that was identified as holding special meaning for participants: “The moving on bit, that's the bit. The future bit. I mean we did the diagnosis and the living with it and what it's like, but now, the last verse…” (**Lisa**).

### Theme 4. we all fit under the same umbrella but we're miles apart: developing a group identity

Analysis of the interview data illustrated that FCGs recognized and respected each other's similarities and differences throughout the songwriting process and that this respect fostered a sense of group identity. Sections of the two different focus groups highlight this. When asked how they found being part of the songwriting group, group one stated:

**Charles:** It also shows that, you know, we're all basically the same group. We all have different problems but they're all the same.

**Lotte:** Yeah, the end result is the same.

**Michelle:** Mm, I also feel that there was a lot of respect between all of us, too. I mean, and when you've got a lot of respect…

**Charles:** And you've come from three completely different angles.

**Michelle:** Yes, yeah.

Group two stated:

**Alison:** Everybody's journey is…

**Lisa:** Aw, totally different. Yeah, yeah. But dementia's got, it's a big umbrella, and there's so many, you know, we all fit under the umbrella but we're miles apart.

Both groups acknowledged the “miles” between each participants' experience of caring for a person living with the dementia and the “completely different angles” and “different problems” of FCGs. However, through respect for and acknowledgement of these differences, participants were able to identify broader commonalities and gain a sense of belonging to the “same group.” **Anne** added that the songwriting group enabled participants to gain a new awareness of other FCGs' struggles and, through this, realize shared thoughts, feelings and experiences despite caring for people with different types of dementia.

**Anne:** I think because you were there with other carers so suddenly you found all these common things…even though they were all different dementias…I guess it all makes us a little more aware of each other and what we're dealing with.

Further, this established sense of group identity enabled participants to suggest a continuation of their meetings *beyond* the group songwriting program. Lotte, Michelle, and Charles articulated that they would like to maintain these supportive connections through continued meetings.

**Lotte:** Well, say if you and I, we get on quite well, if we could meet again that would be lovely [laughing]

**Michelle:** Look, I was going to say, even if they get some morning…do they do that here? Cause I haven't been involved very long. Just to get a few of us together.

**Charles:** The carers.

**Michelle:** Yeah, once a month or something.

### Theme 5. we can stand up for ourselves, we've got stronger: fostering inner strength and personal growth

Four participants (Alison, Lisa, Lotte, and Charles) commented that the group therapeutic songwriting process enabled them to give voice to and/or rethink their personal, social and cultural beliefs and experiences as FCGs of people living with dementia. For some participants, this resulted in a stronger sense of personal resilience and empowerment. For example, **Alison** described the challenges she encountered when taking her husband out to community events, their experience of social exclusion and her fear of social isolation. She highlighted the role of group songwriting in fostering inner strength and resilience, in turn enabling her to cope with these challenges and giving her the confidence to voice her right to participate in the community. She states:

I must admit I was steaming by the end of it. But, I'll pick myself up again. And, yeah, having done this experience [group songwriting] will help me get stronger, get back into it and say, “I've got a right to stay in the community.”

As well as gaining a sense of inner strength from the process of group songwriting, **Alison** stated that the song creation itself “put to words” messages of personal strength, growth, and resilience. In this sense, participants described the song as a personal resource that they could draw on to support their ability to cope with the challenges of caregiving. **Alison** described the personal impact of trying to manage and process her husband's challenging behaviors and how the song helped her “climb out of it”: “I've got better. I've got stronger. And I've got that song running through my head.” Similarly, **Lisa** described the uplifting nature of the “moving on bit” in the song (the last chorus) as influencing her sense of strength and resilience looking into the future: “Here we go! We're off and running and we're going to be okay.”

Enabling people to share their experiences in an open, honest and creative way also helped FCGs to overcome old cultural norms and rethink longstanding personal beliefs and assumptions. **Lotte** and **Charles** dialogued about challenging the “old European way” (Charles) of “grit your teeth and keep going” (Lotte). Both recognized the songwriting group as challenging their assumptions that asking for help was a sign of not coping. They acknowledged the group was helpful in shifting their self-expectations and enabling them to cope.

**Lotte:** I basically grew up very independent. My father got killed when I was 10-months old, as a soldier. My mother, we were refugees, my mother went to work. I was a latch-key kid for most of my young life…and there were lots of things that I just had to do…I never ever asked for help for anything. Because, my mother and I, we were by ourselves…and my mother just said, “whatever needs to be done, it's got to be done now.”

**Charles:** That's the old European way.

**Lotte:** Grit your teeth and keep going. Do the work needs to be done, and, you know. A lot of self-discipline.

**Charles:** I suppose I'm not normally one for, um, what's the word, getting involved in groups. Um, support groups or anything like that. It comes back to what we were talking about earlier. I tend to be self-sufficient.

**Lotte:** Well, I normally am too. But I find now it helps me.

**Charles:** Yeah.

### Theme 6. songwriting groups fill a gap not met by other support groups

FCGs from both songwriting groups found that the songwriting experience was worthwhile and beneficial and addressed an important need not met through other support groups they had attended in the past. Participants described other carer support groups as focusing more on either information sharing or on current day-to-day challenges and issues. This differed from the songwriting group, which allowed FCGs to share their whole carer journey and personal thoughts, feelings and experiences around being a caregiver. Charles talked about how the songwriting group enabled him to “come out” of himself.

**Lotte:** ….The exchange of, and listening to, other people's problems. The carers' morning tea they also discuss a fair bit about how to access help. You know, they had somewhere there from the state trustees as a speaker, for instance. All about…

**Charles:** That's just giving you…

**Lotte:** Giving you information.

**Charles:** As opposed to allowing you to come out.

As opposed to sharing information about the diagnosis and symptoms of dementia or ways to cope with the day-to-day care of someone living with dementia, the songwriting sessions were recognized as addressing what the diagnosis of dementia means for the FCGs themselves. Similarly, these sessions enabled FCGs to not just focus on the negatives and challenges of the caregiver role but to highlight the positives that are sometimes overshadowed by the everyday stress and burden. **Alison** shares:

It's [the songwriting group] certainly got lots of different perspectives from other carers. Probably more than, you know, half a dozen carers sessions. Although you do get something from it [other carer support groups], this drew out some different aspects that hadn't come out of just a normal carers interchange…[The songwriting process] made us look for the positive rather than just the negative. So it wasn't just like in a carer group where you focus on, you know, he's blown up the car again this week or whatever. You know, dealing with just that problem for that particular time. It was trying to look at some of the good things that have come out of it and how we have, as Tony said, learned to just get on with it and deal with those issues.

Participants also highlighted the creative aspect of the songwriting group as a point of difference and something that made the group unique, interesting and valuable. Of the songwriting process, **Anne** stated: “I enjoyed the actual creative process very much…I enjoyed putting the words together and sort of bouncing off other people. It made it more interesting.” Similarly, **Alison** identified the creative aspect of the group as motivation for agreeing to participate: “Um, well for me it was, anything that's creative I've always found is worthwhile and helps my wellbeing…I've always believed that creativity helps you deal with life's problems.” Group two additionally differentiated the songwriting group from other carer support groups by the fact that it was a process with an end product (the song creation), which led to a sense of achievement.

**Charles:** And it's also a very important point, as far as I'm concerned, it's not a meeting that has no end…We've got something to show for the time we spent, as well as everything else.

## Discussion

This feasibility study aimed to determine the participation and retention rates of participants volunteering to take part in a songwriting study, the appropriateness of outcome measures and their sensitivity to change, the estimates of the effects of the intervention on outcome measures, and the acceptability of the intervention. It also aimed to contribute further understandings of how FCGs of PWD experience a group therapeutic songwriting program and generate new knowledge about the processes activated by the intervention protocol.

Participation rates (48%) suggest that some potential participants were either skeptical about the potential relevance or benefits of the project or were not able to commit to attending the sessions at the pre-determined time. Some FCGs who wanted to participate but could not attend, self-selected to the standard care group. Research has indicated that FCGs underutilize a range of resources and support services to cope with the caregiving role (9). This has been associated with carer roles and responsibilities and limited available time and energy (9), as well as the impact of social and cultural norms and self-expectations of the FCG (10). It is well-recognized that FCGs of PWD are time poor (11) and participating in this project may have been perceived as another commitment taking up already short periods of time FCGs have when not directly caring for the PWD. For a large scale trial, careful consideration into how to frame the potential benefits of the project are needed to ensure it is more attractive to already busy FCGs.

Retention (100%) and attendance (91.7%) rates suggested the intervention was perceived as sufficiently engaging and helpful enough for FCGs to return each week and participate in the program. Attendance averages (5.5 of the maximum six sessions) demonstrate commitment in an otherwise busy world of the caregiver (11). When the care-recipient is attending the dementia care day center, it offers FCGs an opportunity to catch up on errands, find space for themselves to rejuvenate and rest, or meet up with other family and friends with the knowledge that they are free from being the “carer” for a few hours in the day. However, participants' choice to come back to the sessions each week suggests that involvement in the therapeutic songwriting program was of more benefit for them than using this time to attend to other activities only possible during periods when they were not directly caring for the PWD. This is supported by the qualitative findings, which indicated participants found the group therapeutic songwriting program worthwhile, helpful, meaningful, and enjoyable. The value and impact of the TSW program is additionally represented in the fact that all interviewed participants stated they would participate in group TSW again.

**Figure 2:**
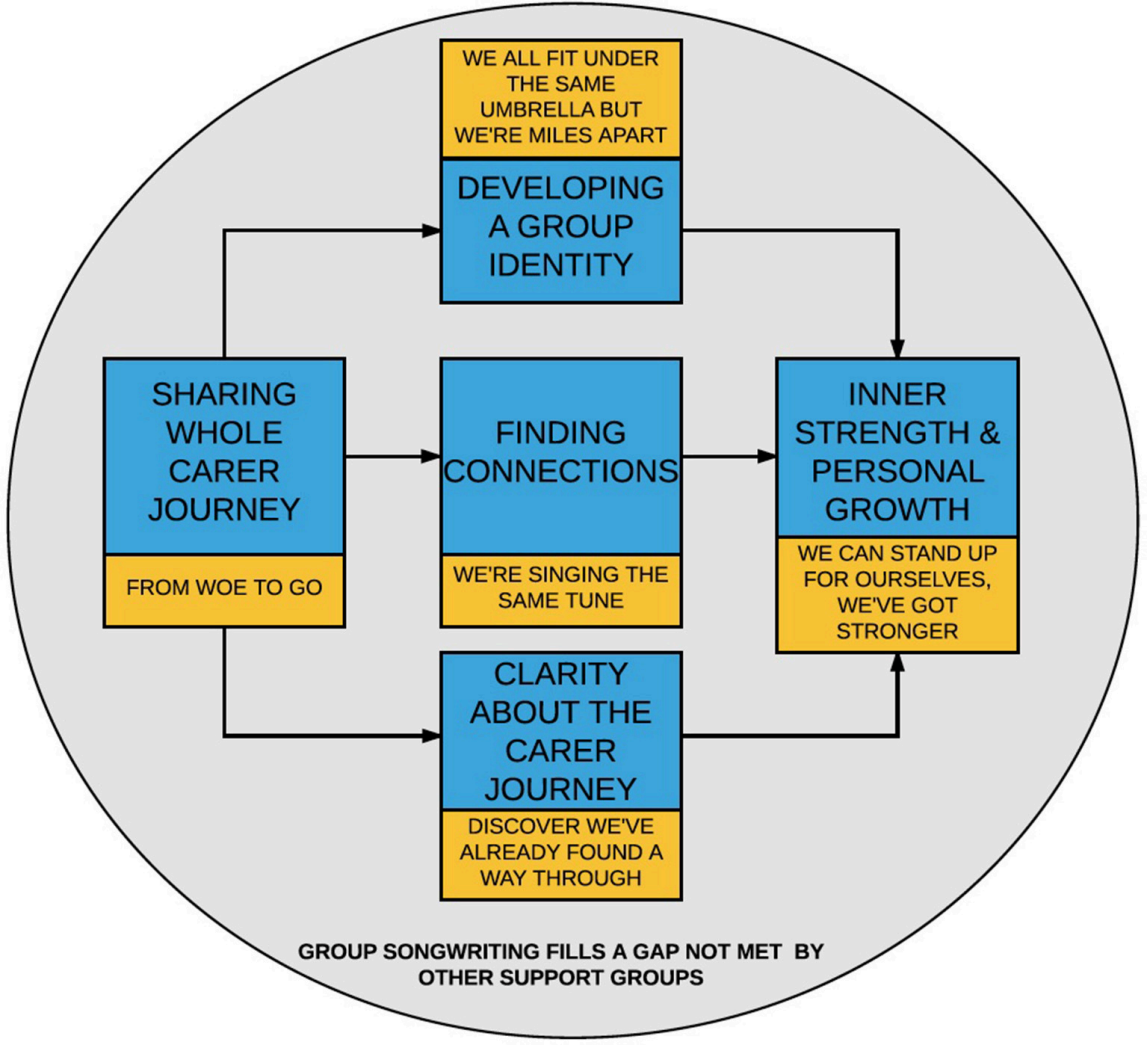
Processes activated by the group songwriting experience.

A moderate effect size was observed for differences between pre- and post-scores on the primary outcome measure (PHQ-9) measuring depression for the Experimental Group, while smaller effect size was found for the Standard Care group. The existence of this observed effect, combined with the findings from focus group analysis (see Figure 2 and discussion below), suggests that depression would be worth measuring in a fully powered trial. The impact of songwriting on depression has been reported in other studies with people with acquired brain injury and spinal cord injury (40), people on an acute care psychiatric unit (51), people undergoing detoxification (52), and people undergoing treatment for cancer (53). This suggests there may be similar underlying mechanisms associated with reductions in levels of depression as a consequence of therapeutic songwriting.

Neither of the secondary outcomes measuring the quality of the caregiving relationship and experience of caregiving (QCPR, PACQ) demonstrated any differences over time. Qualitative data indicated that FCGs' coping might be a more relevant measure of change to caregiver wellbeing than examining the quality of the relationship, especially as the intervention was tailored to enhance both emotion-focused and problem-focused coping. The influence of this, and in turn relevance of coping, is reflected in themes suggesting that the songwriting process enabled participants to share their whole caregiver journey (theme 1), including the “emotional journey,” and develop important supportive connections with other FCGs (theme 2). Participants' description of the group as an opportunity for emotional disclosure and garnering support from other FCGs is supported by Brotons and Marti's (22) study on the effects of music therapy-oriented support groups for FCGs. More specifically, our findings regarding the impact of group TSW on FCGs sense of connection, belonging and group cohesion is supported by previous studies on TSW with PWD (33, 34) and older adults (35). Further, qualitative analysis highlighted that FCGs gained clarity and insight into their caregiver journeys (theme 3), and developed inner strength and personal growth to cope with their caregiver role (theme 5). This occurred both through the songwriting process and by using their song creation as a personal resource, and may have had a flow-on effect to the reductions in depression noted in our quantitative data. These findings are supported by the literature on group TSW with FCGs of PWD (36, 37). These studies found the songwriting process fostered healthy coping skills (36), and enabled participants to gain insight into themselves, each other and their caregiver context (36, 37).

Our qualitative data analysis further supports the acceptability of the intervention in fulfilling a much needed gap not met by other support groups (theme 6). Figure 2 illustrates how the emergent themes interconnect and explains the therapeutic process experienced by the FCGs. Through sharing their individual experiences of caregiving (theme 1), FCGs were able to identify their collective experiences within the group, leading to the development of connections with one another (theme 2), and a supportive group identity (theme 4). These connections, support and sense of group identity served as a source of strength for the FCGs; a coping tool. Similarly, by offering the opportunity for FCGs to share and explore their individual carer journeys (from “woe to go”), the therapeutic songwriting process enabled FCGs to gain insight into their own thoughts, feelings, and experiences around caregiving. Through the songwriting process, FCGs were able to recognize their own strengths and positive aspects of the caregiver experience, affirming their ability to cope and manage within their caregiver role (theme 3). This affirmation that the FCGs are on the right path or have already “found a way through” further reinforces inner strength, resilience, and personal growth (theme 5).

## Limitations

This feasibility study was a simple pre-post test design and participants were able to choose to participate in either the Experimental Group or Standard Care condition. It is not clear what the retention rates would have been if people were not free to self-select to condition. For example, attendance among the experimental group was high, but uptake was rejected by the standard care group. It is unclear whether there would have been more dropouts had those initially preferring to be in the experimental group were randomized into the standard care group and vice versa.

Authors 1 and 2 who were responsible for delivering the intervention and collecting data were not blinded to condition and while this was considered imperative to the qualitative data analysis, it violates best research practice for collecting quantitative data. Although the researchers emphasized the need for participants to respond in an honest way, participants may have felt pressure to respond in a certain way due to the relationship developed with authors 1 and 2. Further, small sample sizes and non-homogeneous groups mean the interpretations of the findings should be viewed with caution. For example, the relationship of the caregiver to recipient varied from spouse, to sibling, to adult child. These inherently different caregiver-care recipient relationships would undoubtedly lead to different types of stressors and issues of concern, as well as impacting on the types of relationships and connectedness felt between the caregivers in the group (36).

In addition, while information was provided on participants' age, gender, and caregiver/care recipient relationship, other factors influencing the caregiver experience were not incorporated into the study design. Family caregivers' history and experience of caregiving was explored in the experimental group during the therapeutic songwriting process, however, this information was not formally collected from participants across both conditions. For example, relevant information may include participants' length of time as a FCG, previous history of caring, existing family structure and support, external support (e.g., home care package), and attendance of other carer support groups. Future studies may wish to collect and consider additional participant data in order to gain further understanding of factors impacting the caregiving experience and, in turn, the experience of the intervention.

Finally, while the focus group can be an effective medium for exploring issues in depth (54), richer data might have been generated if all participants had been interviewed individually. Future research would benefit from taking the themes generated from this study, and testing and refining the emerging theory through individual interviews with FCGs participating in similar group songwriting interventions.

## Conclusion

An examination of songwriting with a small sample size appeared to be meaningful to participants as indicated by their qualitative data. Depression appears to be a relevant variable to study as indicated by the effect sizes obtained from this very small sample. Similarly the PHQ-9 is useful to capture subtle changes in levels of depressive symptoms. The other measures were not useful to capture changes in this context. The large observed effect size on a measure of depression suggests that this area may be worth examining in a fully powered trial. Qualitative data suggests coping may be a more meaningful variable to test in future studies. The qualitative analysis revealed that songwriting enabled caregivers to share their journeys of caregiving in a holistic way, and through doing so, enabled them to feel more connected, develop a supportive group identity, clarify their perceptions of the caregiver journey, and develop inner strength and personal growth that would enable them to cope with their caregiving role.

## Ethics statement

This study and study protocol was carried out in accordance with the recommendations of National Health and Medical Council Ethical guidelines, The University of Melbourne & Human Research Ethics committee approved the project approval number 1748699 with written informed consent from all subjects. All subjects gave written informed consent in accordance with the Declaration of Helsinki.

## Author contributions

FB was responsible for the overarching design of the study. FB designed the intervention, conducted qualitative focus groups, undertook the qualitative analysis, and oversaw the delivery of the interventions. PS-S provided the interventions, facilitated quantitative data collection, conducted focus group interviews, and co-contributed to the qualitative analysis. IC conducted the quantitative analyses. JT and Y-EL selected the appropriate outcome measures and advised on administering the measures. All authors contributed to the writing of the manuscript.

### Conflict of interest statement

The authors declare that the research was conducted in the absence of any commercial or financial relationships that could be construed as a potential conflict of interest.
